# Gene Therapy With Regulatory T Cells: A Beneficial Alliance

**DOI:** 10.3389/fimmu.2018.00554

**Published:** 2018-03-19

**Authors:** Moanaro Biswas, Sandeep R. P. Kumar, Cox Terhorst, Roland W. Herzog

**Affiliations:** ^1^Division of Cellular and Molecular Therapy, Department of Pediatrics, University of Florida, Gainesville, FL, United States; ^2^Division of Immunology, Beth Israel Deaconess Medical Center (BIDMC), Harvard Medical School, Boston, MA, United States

**Keywords:** regulatory T cells, tolerance, gene therapy, chimeric antigen receptor regulatory T cells, adoptive transfer, cell therapy, adeno-associated virus vectors, lentiviral vectors

## Abstract

Gene therapy aims to replace a defective or a deficient protein at therapeutic or curative levels. Improved vector designs have enhanced safety, efficacy, and delivery, with potential for lasting treatment. However, innate and adaptive immune responses to the viral vector and transgene product remain obstacles to the establishment of therapeutic efficacy. It is widely accepted that endogenous regulatory T cells (Tregs) are critical for tolerance induction to the transgene product and in some cases the viral vector. There are two basic strategies to harness the suppressive ability of Tregs: *in vivo* induction of adaptive Tregs specific to the introduced gene product and concurrent administration of autologous, *ex vivo* expanded Tregs. The latter may be polyclonal or engineered to direct specificity to the therapeutic antigen. Recent clinical trials have advanced adoptive immunotherapy with Tregs for the treatment of autoimmune disease and in patients receiving cell transplants. Here, we highlight the potential benefit of combining gene therapy with Treg adoptive transfer to achieve a sustained transgene expression. Furthermore, techniques to engineer antigen-specific Treg cell populations, either through reprogramming conventional CD4^+^ T cells or transferring T cell receptors with known specificity into polyclonal Tregs, are promising in preclinical studies. Thus, based upon these observations and the successful use of chimeric (IgG-based) antigen receptors (CARs) in antigen-specific effector T cells, different types of CAR-Tregs could be added to the repertoire of inhibitory modalities to suppress immune responses to therapeutic cargos of gene therapy vectors. The diverse approaches to harness the ability of Tregs to suppress unwanted immune responses to gene therapy and their perspectives are reviewed in this article.

## Introduction

Gene therapy has the tremendous potential to completely cure with a single treatment, diseases previously classified as untreatable, or disorders that could be managed but not corrected. Correction is achieved by transferring a functional copy of a gene, which is otherwise mutated in the diseased state, or by editing the defective gene in the patient’s body. After a period of major setbacks during the late 1990s and early 2000s, this technique has reemerged as a major breakthrough in regenerative medicine (1, 2). A clear proof of clinical efficacy has mostly been observed in ocular diseases (inherited blindness), primary immune deficiencies, beta-hemoglobinopathies, and more recently hemophilia (2–9). Approaches for gene therapy in the clinic are based on *in vivo* delivery to post-mitotic cells or tissues, or *ex vivo* delivery into autologous hematopoietic stem cells (HSCs), followed by reinfusion into the patient. Treatment of blindness by *in vivo* gene transfer (NCT00999609 and NCT00516477) is the first representative gene therapy drug approved in the USA by the Food and Drug Administration (Luxturna, Spark Therapeutics). In the meantime, cancer gene therapy drugs have already been approved, which include the virotherapeutic Imlygic (an engineered oncolytic Herpes virus, Amgen), chimeric antigen receptor (CAR) T cell therapy such as tisagenlecleucel-T (Kymriah, Novartis), and most recently, axicabtagene ciloleucel (Yescarta, Kite Pharma). The latter are of particular significance for this review, as they underscore the potential for therapies based on genetically engineered T cells.

## Immune Responses to Gene Therapy

The aim of successful gene therapy is the safe and effective delivery of the replacement gene at therapeutic levels, potentially for the lifetime of an individual. A key obstacle to successful gene therapy is the host’s immune response to both the viral vector and the transgene product. A fatal inflammatory immune response to the adenoviral vector almost brought the field to a stop in 1999 in a gene therapy clinical trial (10), although the safety and efficacy of gene therapy has been clearly established since then.

Gene therapy by vector administration into immune-privileged sites like the brain, eye, and testis has successfully achieved long-term transgene expression (11, 12). However, vector-mediated delivery into immune-competent organs is complicated by prevailing neutralizing antibodies that can limit the efficacy of transduction in patients (13). Although initial trials enrolled patients after a very careful selection process, gene therapy is becoming more common, and patient inclusion criteria are expected to be less exclusive, likely including patients with prevailing neutralizing antibodies or cross-reactive immunologic material- negative mutations.

At present, several viral vectors have been established as vehicles for gene transfer. Common among these are adenoviral vectors, gamma retroviral vectors, adeno-associated virus (AAV) vectors, and lentiviral vectors (LVs). For LV, gene therapy has been clinically approved for *ex vivo* gene transfer (14, 15), and the use of LVs for *in vivo* gene replacement is being evaluated in preclinical models (16, 17). This is facilitated by the low prevalence of neutralizing antibodies to LVs and the capacity to accommodate larger gene inserts. The new generation of replication-deficient vectors is gutted and nonpathogenic. Unlike gamma-retroviruses that favor integration near transcription start sites, LVs have been shown to integrate into active genes, making the chances of insertional mutagenesis and clonal expansion less likely (18). Potential innate and adaptive immune responses, which vary in magnitude, can develop toward the encoded transgene (19), envelope pseudotype or proteins acquired during the packaging process (20). LV-triggered innate immune responses such as type I IFN are primarily mediated by viral genome engagement with TLRs, possibly TLR9 and TLR7 (21–23).

Cytotoxic T lymphocyte (CTL) responses to both viral antigen and transgene have been observed with early-generation adenovirus and in preclinical models of *in vivo* adenoviral gene transfer (24–26). Replication-deficient, first- and second-generation adenovirus vectors are now being used in cancer gene therapy clinical trials, particularly for solid cancers (NCT01811992, NCT02630264, NCT01310179, NCT00870181 and NCT01147965). The high immunogenicity of adenoviral vectors has also made them attractive candidates as vaccine carriers. For example, the recent devastating outbreak of Ebola prompted a rapid phase I clinical trial of the replication-defective, chimpanzee adenovirus type 3-vectored Ebola virus vaccine (cAd3-EBO) (27). There is interest in helper-dependent or gutless third-generation adenoviral vectors, because of reduced *in vivo* immune responses as compared to first- and second-generation adenoviral vectors (28). However, innate immune responses are still elicited (29).

For *in vivo* gene delivery, recombinant AAV is the vector of choice due to its ease of construction, wide tissue tropism, and presumed lack of pathogenicity as it does not efficiently transduce macrophages, mature DC, and other antigen-presenting cells (APCs), although endocytosis of AAV has been associated with innate immune activation (30). It has been shown that the TLR9–MyD88 pathway is crucial for cross priming AAV capsid-specific CD8^+^ T cells, a process that requires the cooperation of both pDC and cDC subsets of dendritic cells, as well as for activating transgene-specific CD8^+^ T cell responses (31, 32). Anti-capsid effector T cell responses have been elicited in trials where the vector was administered outside of the retina or CNS. These have been shown to be responsible for deleterious immune responses against transgene-expressing cells, affecting therapeutic efficacy (33–36). Anti-capsid effector T cell responses were not predicted by preclinical studies, highlighting one of the major preclinical challenges when working with AAV (34).

## Treg Types and Characteristics

The molecular characteristics that enable Tregs to modulate the activation of responder T cells render them uniquely suitable to limit immune responses to a therapeutic gene. Tregs have potent immunosuppressive properties that can be harnessed to confer antigen-specific immunomodulation in a therapeutic setting (37). Treg activity is required to maintain immune homeostasis in the presence of autoreactive T cells. Thus, they have defined roles in diverse clinical conditions including cancer, autoimmune disease, and transplant rejection (38–40). The most commonly studied among them are the CD4^+^CD25^+^FoxP3^+^-expressing Treg subset, which are thymus-derived and called thymic, natural, or central Tregs (41). Natural Tregs commonly exhibit specificity to self-antigen and are essential for maintaining tolerance to self-tissues. Treg cells derived from outside the thymus are often referred to as induced, adaptive, or peripheral Tregs. These can be antigen-specific effector T cells induced to express FoxP3, or type 1 Treg (Tr1) cells that are FoxP3^−^, express surface LAG-3 (CD223) and CD49b, and secrete IL-10 (42, 43). A recent FoxP3^−^ subset, with a regulatory activity, expressing latency-associated peptide (LAP) on the surface as latent TGF-β complexes has also been defined, which can be cleaved to release active TGF-β (44). Nonetheless, there is scientific consensus that each Treg subset has suppressive capacity and is integral to maintaining tolerance, as has been observed in treatment for autoimmune diseases and in gene therapy.

Resting natural Tregs are usually CD25^hi^, CD127^−^, L-selectin (CD62L)^+^, CTLA-4 (CD152)^+^, and ICOS^±^. Other natural Treg markers such as neuropilin are specific for mice (45, 46). The activation of both natural and peripherally induced Tregs (iTreg) is associated with inducible upregulation of markers, many of which are not Treg exclusive, but are common in activated effector and memory T cells. These include but are not limited to CD69, CD25, CD44 (47, 48), CD39, and CD73 (49), galectin-1 and -10 (50, 51), glycoprotein A repetitions-predominant (GARP) and LAP (52, 53), CTLA-4 (CD152) (54), Ki67, GITR (TNFRSF18), TNFR2, and ICOS (55). In particular, CTLA-4 has been found to regulate many aspects of Treg suppression and can control the progression of autoimmune disease (56–59). In some cases, activated Tregs have been associated with an increase in FoxP3 expression (60–62).

Regulatory T cell suppressive function has been shown to be primarily TCR contact-dependent. TCR signaling is crucial for Treg development, differentiation, and suppressive function (63). Tregs use multiple mechanisms to suppress immune responses, depending on the nature and tissue-specific location of the antigen (lymphoid and non-lymphoid tissues). These include antigen-specific and bystander suppression associated with the release of cytokines such as IL-10, TGF-β, and IL-35 (64), IL-2 deprivation, direct cell killing (65), the production of metabolic intermediates (66, 67), and the modulation of dendritic cell maturation and stimulatory function (68, 69).

## Evidence that Tregs Limit Immune Responses in Muscle Gene Transfer

Gene delivery into the muscle is attractive as a potential source for therapeutic protein expression. Muscle gene therapy is ideal for degenerative disorders like the muscular dystrophies, storage disorders leading to metabolic myopathy such as Pompe disease or for the production of enzymes like alpha-1 antitrypsin (AAT) (70). A major barrier to muscle gene transfer is the need to deliver the replacement gene body wide, necessitating multiple injections into various sites throughout the body, thereby increasing the potential for inflammatory immune responses (71, 72). Further, the often-required high vector doses also enhance the risk of provoking an immunological response. Physiologically, delivery into the muscle environment causes inflammation, presumably due to the high frequency of resident macrophages.

Clinical trials using muscle gene transfer have shown promise for many disorders, some of which show very poor prognosis with conventional therapy. For example, gene replacement therapy is a feasible approach for the treatment of the lysosomal storage disorder, Pompe disease, which particularly affects the skeletal and cardiac muscle, and neural tissues. Initial clinical experience in Pompe disease shows that the direct delivery of AAV1–hGAA into the diaphragms of affected children is safe, well tolerated, and efficacious (73, 74). Neutralizing antibody development against the hGAA transgene product and the viral vector prevents therapeutic efficacy and vector readministration, respectively (75, 76). Interestingly, T cell reactivity toward the vector has not been observed to date (73). Preclinical data show that lentiviral correction of HSCs by *ex vivo* transduction was effective in ameliorating Pompe disease in a mouse model (77), which could be a viable alternative for preventing immune responses by facilitating central tolerance.

The detection of T cell responses to the capsid in peripheral blood mononuclear cells is not always associated with a deleterious immune response, as seen during gene transfer trials with AAT. Despite the detection of T cell reactivity against the vector and infiltrates into the treated muscle, the transgene was still expressed in subjects who received an AAV1 vector encoding for AAT (78–81). Interestingly, CD4^+^CD25^+^FoxP3^+^ Tregs were found within the infiltrating cells (~10%) in vector-injected muscle and were associated with a time-dependent decrease in muscle inflammation, which may have prevented the destruction of transduced myofibers (82).

Similarly, a population of Tregs was shown to accumulate in muscles of dystrophic mice and in muscle biopsies from Duchene muscular dystrophy (DMD) patients (83, 84). These IL-10-secreting Tregs improved the dystrophic phenotype by decreasing inflammation associated with the disease, and their depletion resulted in worsening of the disease phenotype. Therapeutic targeting of Tregs with IL-2/anti-IL-2 complexes had a beneficial effect of reducing muscle inflammation and injury in dystrophic mice. Thus, these observations demonstrate the potential of Treg-modulating agents to induce a local Treg population in muscle at the time of gene transfer to reduce muscle inflammation and favor the maintenance of transgene expression in DMD. Another feasible alternative is adoptive immunotherapy with polyclonally expanded or antigen-specific Tregs at or during the time of gene therapy. In an earlier pivotal study, the administration of exogenous transgene-specific Tregs concomitantly with AAV gene transfer was shown to lower anti-transgene immune reactivity and allow stable transgene expression in normal muscle (85). This established that adoptively transferred CD4^+^CD25^+^ regulatory T cells can induce a sustained transgene engraftment in solid tissues. Combinatorial treatments using adoptive Treg transfer as adjunct therapy may thus enhance the therapeutic effect of gene delivery by developing tolerance toward the gene delivery vehicle or transgene product.

## The Liver as an Ideal Site for Immune Regulation

The administration of gene therapy systemically leads to rapid accumulation of high levels of vector particles within the liver. Specialized liver-resident cells mediate the “liver tolerance effect,” which establishes local and systemic tolerance to self and foreign antigens. This has been attributed to the expression of inhibitory cell surface ligands for T cell activation and the production of anti-inflammatory mediators (86).

The utilization of liver tropic viruses, engineered vector serotypes, and liver-specific promoter and enhancer elements have improved liver gene delivery and increased gene expression to clinically therapeutic levels (87). Much of the present interest in the development of liver-directed gene therapy stems from recent clinical success in treating the X-linked coagulation disorder hemophilia B, with restoration of clinical levels of factor IX (FIX) to hemophilia B patients for sustained periods greater than 5 years (7, 36). A transient increase in liver enzymes, presumably due to the reactivation of a memory CTL response to the vector, was earlier observed, although intervention with corticosteroid administration at the first sign of hepatocellular injury could halt the increases in liver enzymes and sustain FIX expression (34). Similarly, high endogenous levels of clotting factor have been reported in recent clinical trials for both hemophilia A and B (88, 89).

The development of inhibitory antibodies that neutralize factor VIII (FVIII) or FIX is a major complication of protein replacement therapy as well as gene therapy for patients with hemophilia (90). Preclinical studies in small and large animal models of hemophilia have demonstrated that gene therapy strategies and the continuous exposure to clotting factor can promote tolerance and eradicate preexisting antibodies (91–94). Nonetheless, there is still a risk of developing neutralizing antibodies to the coagulation factor product following hepatic gene transfer (95). There is strong evidence that Tregs are an important element of the mechanism by which self-tolerance is maintained and inhibitor development, a T helper-dependent response, is prevented (96–99). In many cases, immune tolerance to hepatic gene transfer of hFIX has also been associated with the induction of Tregs (100–102). We propose that the adoptive transfer of Tregs in the setting of liver gene therapy has the potential to avoid the general immunosuppression that many corticosteroid drugs pose, instead favoring tolerance to the transgene in an antigen-specific, safe, and transient manner.

Another field where liver gene therapy has garnered interest is in the treatment of autoimmune disorders like rheumatoid arthritis, multiple sclerosis, and type 1 diabetes (T1D). Replacement gene delivery in these cases is complicated by the development of an immune response to the therapeutic gene. Studies have demonstrated that gene therapy into the tolerogenic liver microenvironment can abrogate the development of experimental autoimmune encephalomyelitis (EAE) even if the target antigen for the inflammatory T cell response is in a distant organ, such as the central nervous system (103, 104). Protection from EAE was dependent on the induction of antigen-specific CD4^+^CD25^+^Foxp3^+^ Tregs (103, 104). Using the same principle in a preclinical mouse model T1D, Akbarpour et al. showed that targeting LV-mediated insulin gene expression to hepatocytes induced regulatory T cells specific for insulin, which halted immune cell infiltration into the pancreatic islet and protected from T1D (105). Thus, it appears that targeting gene transfer to hepatocytes can favor the induction of antigen-specific Tregs systemically, making the liver an attractive target for achieving transgene tolerance.

## *In Vivo* Tolerance Induction with Treg

Given the critical role of Treg in maintaining immune regulation of transgene-specific responses, an obvious treatment of choice is the *in vivo* induction of antigen-specific Treg by a specific or a combination drug treatment. Global immune suppression by steroid or chemotherapeutic drugs, while beneficial when given transiently, does not have the advantage that a more targeted and a lasting transgene product-specific Treg response can offer. One method of inducing Treg is to coadminister the antigen with the macrolide immunosuppressant rapamycin (sirolimus), which inhibits cell cycle progression of activated T cells by mTOR pathway blockade, leading to T cell anergy or deletion (106). At the same time, the inhibition of the T cell stimulatory activity of dendritic cells (107) and mTOR-independent signaling by Tregs (108) result in the enrichment of antigen-specific CD4^+^CD25^+^FoxP3^+^ Treg (109–111). This effect can be enhanced by the addition of cytokines such as IL-10 or Flt3L, which have been shown to promote tolerance in protein replacement therapy (96, 99). Prophylactic therapy of IL-10 in combination with rapamycin and antigen has also been successful in the prevention and reversal of inhibitory antibody responses in muscle gene transfer of therapeutic FIX in hemophilia B mice (96, 99, 112). Likewise, the introduction of rapamycin with liver gene therapy resulted in a markedly enhanced expression of human acid-α-glucosidase in nonhuman primates, likely due to the induction of hepatic autophagy and is being evaluated for readministration of the AAV vector (113).

Tolerance to antigens administered by the oral route is another approach to inhibit antigen-specific immune responses by targeting the gut-associated lymphoid tissue (114). Multiple immune cell types have been shown to be involved in mediating this state of non-responsiveness, including gut resident dendritic cells, FoxP3^+^ Tregs and CD4^+^CD25^−^LAP^+^-expressing Tregs (115–117). Gut homing receptors and cytokines such as TGF-β and IL-10 have been shown to be responsible for the infiltration/differentiation/local expansion of these Treg subtypes and the induction of tolerance (118). Significantly, oral tolerization improved long-term transgene persistence and expression as shown in a recent study using AAV-mediated gene transfer of the model antigen OVA (119).

## Cellular Therapy with Treg

Extensive preclinical studies have demonstrated that Tregs play a key role in both the induction and maintenance of tolerance. Adoptive immunotherapy with autologous or donor Tregs has shown promise in several clinical trials for autoimmune disorders and in transplant conditions (120, 121). With new GMP protocols in place, FoxP3^+^ Tregs can undergo polyclonal or antigen-specific expansion with high purities (122, 123). Protocols to generate donor-specific Tr1 cells are also well established (124–126). Clinical trials with freshly isolated or *ex vivo* expanded FoxP3^+^ (127–130) or Tr1 cells (ALT-TEN trial) (131) (from umbilical cord blood or peripheral blood) as a cellular therapy given at or shortly before/after transplantation have been carried out for tolerance to graft-versus-host disease (GvHD) in patients undergoing allo-HSCT for hematological malignancies. Alternatively, ultra-low-dose (ULD) IL-2 has been suggested to selectively expand nTreg *in vivo*, suppressing alloreactive responses in GvHD prophylaxis and treatment (132–134). Supplementing Treg infusion with ULD IL-2 to promote Treg persistence and survival for the treatment of onco-hematological diseases is being tested (NCT02991898).

Studies testing the safety and feasibility of autologous polyclonal or alloantigen-specific Treg infusion for conferring tolerance in solid organ transplantation are currently ongoing [NCT02145325 (TRACT), NCT02088931, NCT02711826 (TASK), UMIN-000015789] (135). The ThRIL study (NCT02166177) has been initiated to evaluate the efficacy of Treg cell therapy, in combination with immune-suppressive drugs, in liver-transplant recipients.

The ONE Study (www.onestudy.org) is a phase I/IIa clinical trial aimed at testing the safety and feasibility of seven different regulatory T cell populations in living donor kidney transplants. This multicenter study compares autologous *ex vivo* expanded polyclonal CD4^+^CD25^+^ nTregs from peripheral blood, Tr1 cells, donor alloantigen-driven Tregs (darTreg), and alloantigen-driven T cells anergized by costimulation blockade, tolerogenic dendritic cells, and regulatory macrophages (Mregs). Comparisons will be made between patients receiving standard immunosuppressive treatment (basiliximab followed by tacrolimus, mycophenolate mofetil, and prednisolone) and those receiving immunotherapy (136).

Finally, Treg therapy has also been applied to autoimmune and inflammatory disorders (e.g., TRIBUTE trial for Crohn’s). An autologous antigen-specific Tr1 therapy for refractory Crohn’s disease is in development (Ovasave, Txcell). In a study of pediatric patients with T1D, single and repeat infusions of polyclonal expanded Tregs were found to be safe and effective in patients (137). As with GvHD, the effect of low-dose IL-2 on *in vivo* induction of Tregs for 12 autoimmune and inflammatory diseases is being tested in a multicentric trial (TRANSREG, NCT01988506). Moreover, studies to assess the safety of Treg immunotherapy supplemented with IL-2 and the persistence of infused autologous Tregs in patients with recent onset T1DM are being undertaken (TILT study, NCT02772679) (138). In an earlier report by the same group, a study of 14 adult subjects with T1D who received *ex vivo* expanded polyclonal Tregs saw a subset of Tregs remaining in circulation at 1 year after transfer (139).

Taken together, these studies reveal that infusions of *ex vivo* expanded FoxP3^+^ or FoxP3^−^ (Tr1) cells are safe, well tolerated, and can aid in tolerance in many inflammatory and autoimmune conditions.

## Supplementing Gene Therapy with Treg Adoptive Transfer

The immune suppressive properties of Tregs have generated interest in utilizing this cell population for tolerance toward the transgene product. Not only are Tregs critical for establishing central tolerance during development and in preventing autoimmunity, they are also involved in inducing tolerance toward exogenous antigens, such as therapeutic proteins. Ideally, immune modulation to suppress vector or transgene-specific responses should eliminate undesired immune cells while sparing protective immunity.

There is ample evidence that adoptive immunotherapy with polyclonal or engineered Tregs can improve protein replacement therapy in inherited protein deficiencies (98). On the other hand, very few studies on infusing Tregs to improve tolerance to gene therapy have been carried out. So far, gene therapy into immune privileged sites like the eye has not been associated with a deleterious immune response. Likewise, gene delivery into tolerogenic organs, particularly the liver, has in fact been shown to induce Tregs *in vivo*. However, liver-directed gene therapy, while successfully diminishing immune responses toward the transgene product, does not completely eliminate the development of cytotoxic T cells that can subsequently lead to the potential immune-mediated deletion of transgene-expressing cells (140). Similarly, although the development of neutralizing antibodies to the transgene product has so far not been observed in the small number of liver gene therapy clinical trials in humans, the possibility remains a concern as observed in preclinical studies for the immunogenic FVIII molecule (140, 141) (unpublished observations). Strategies such as using microRNA target sequences (miR-142-3p) in the LV to de-target transgene expression from professional APCs, coupled with restricted expression in either hepatocytes or liver endothelial cells, have led to improved transgene expression. This has been shown to correlate with the emergence of transgene-specific Tregs, which induced tolerance in preclinical models of hemophilia A and B (17, 142, 143).

**Figure 1:**
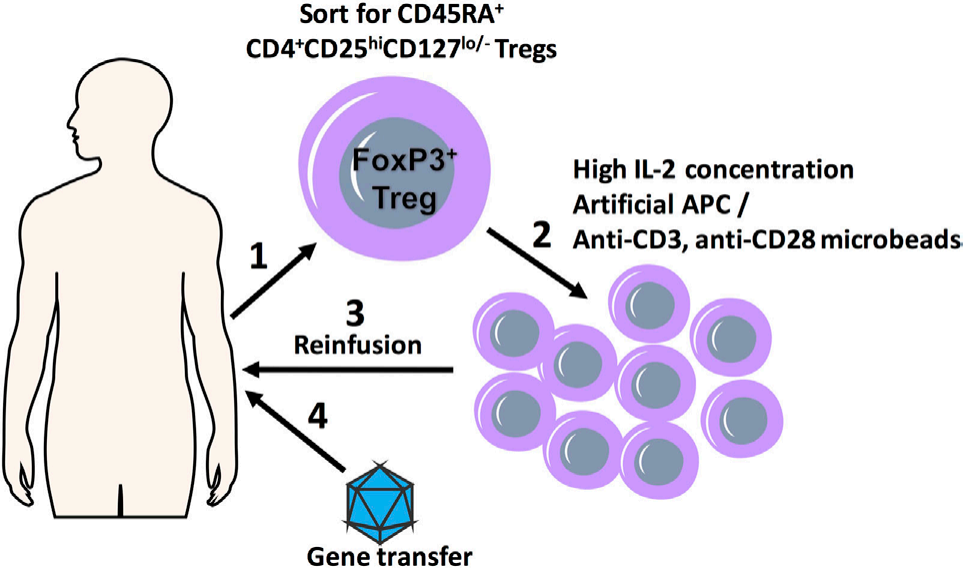
A scheme detailing combination regulatory T cell (Treg) adoptive therapy with gene transfer for tolerization of immune responses. FoxP3^+^ Treg cells with polyclonal specificity are harvested from the patient (1) and *ex vivo* expanded in the presence of high IL-2 concentrations and artificial APC (aAPC) or anti-CD3, anti-CD28 microbeads using GMP protocols (2); expanded Tregs are transplanted back into the patient (3), which is followed shortly by gene transfer (4).

**Figure 2:**
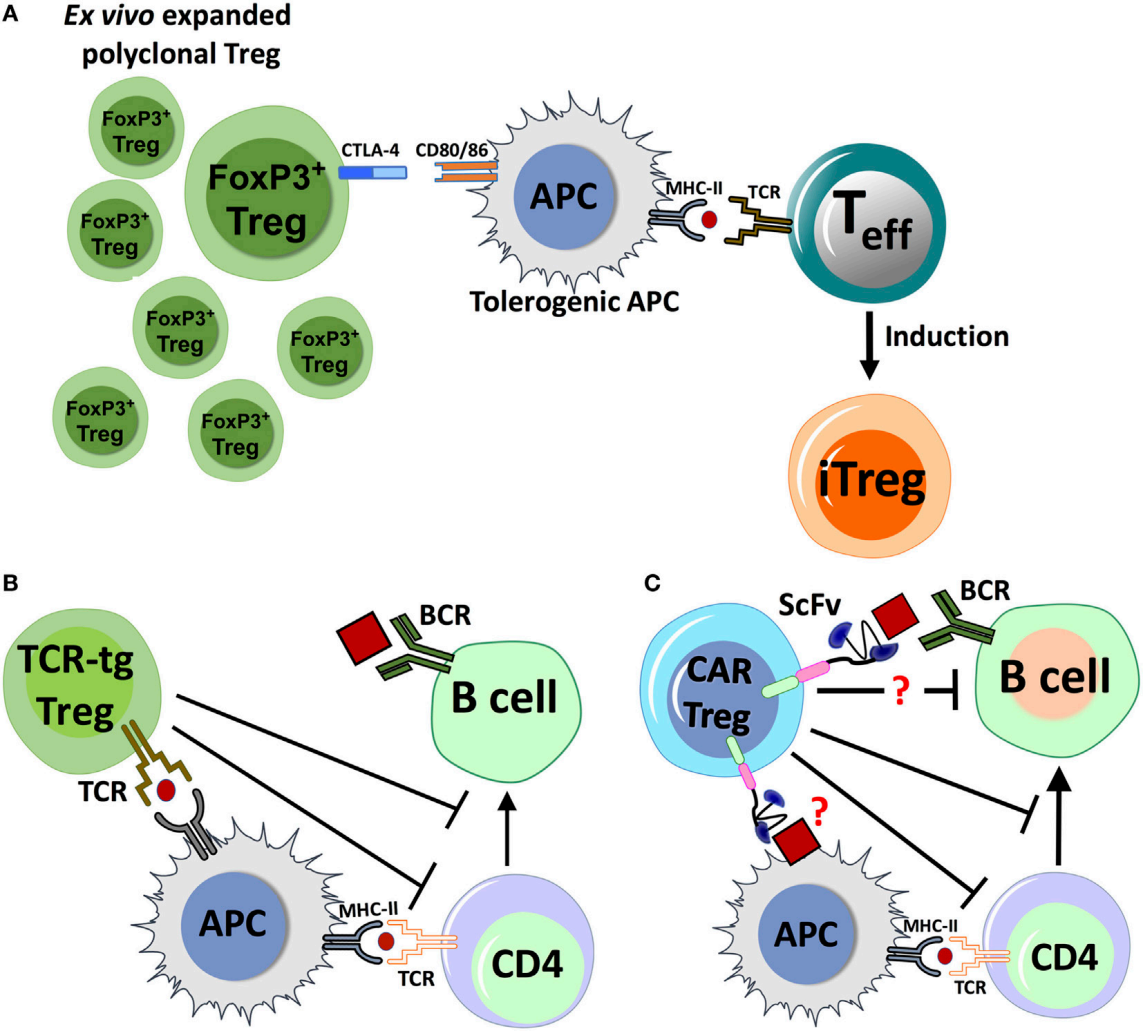
Proposed mechanisms for immune suppression by adoptive transfer of polyclonal FoxP3^+^ regulatory T cell (Treg), chimeric antigen receptor (CAR)-Treg or TCR-transgenic (TCR-tg) Treg. **(A)** Adoptively transferred *ex vivo* expanded Treg with polyclonal specificity can interact with antigen-presenting cell (APC). Inhibitory receptors like CTLA-4 can compete with the costimulatory molecule CD28 to bind to CD80/86 receptors, and combined with other factors, it can lead to APC tolerization. Tolerogenic APCs interact with activated antigen-specific T effector (T_eff_) cells, which leads to conversion of T_eff_ to induced Treg (iTreg). **(B)** Natural Treg engineered with TCR specificity for antigen (TCR-tg Treg) can recognize antigen presented by APCs, directly suppressing the APC’s capacity to costimulate T_eff_ cells. TCR-tg Treg can also directly inhibit CD4^+^ T helper cells, which in turn affects T cell help to antigen-specific B-cells. **(C)** Putative mechanisms for antigen recognition and suppression by CAR-Treg. CAR-Tregs may recognize either a B-cell bound antigenic epitope or antigen on the surface of APC, which can trigger the activation and proliferation of the CAR-Treg through transmembrane and intracellular-signaling domains. The mechanisms by which CAR-Tregs exert their suppressive effects are not clearly defined, but may include interactions with key cell types.

In an earlier study on complementing gene therapy with Treg adoptive transfer, Gross et al. established that the injection of influenza hemagglutinin (HA)-specific CD4^+^CD25^+^ Tregs, concomitant with gene transfer, enabled persistent HA transgene expression in the muscles of mice (85). Cytotoxic T cell responses, as well as circulating anti-IgG antibodies to HA, were impaired in HA-Treg recipients. These findings were applied to a disease setting for hemophilia A, where nonviral gene transfer of the therapeutic FVIII plasmid resulted in supraphysiological levels of FVIII, but triggered inhibitory antibody development and loss of functional FVIII activity. Adoptive transfer of cells enriched for FVIII-specific Tregs into naïve hemophilic mice, followed by plasmid challenge, led to a significantly diminished inhibitory antibody formation for a prolonged period, as compared to control animals (144). These studies establish the potential of Tregs to modulate immune responses to the transgene product in an antigen-specific manner (Figure 1). Our group added to these initial studies by demonstrating that adoptively transferred *ex vivo* expanded Treg could be used to improve gene therapy of FIX in a mouse model of hemophilia B (98). In the study, polyclonal *ex vivo* expanded autologous CD4^+^CD25^+^FoxP3^+^ Treg administered at doses similar to those currently used in clinical trials (~5 × 10^7^ cells/kg) was able to prevent the formation of an adaptive immune response in hemophilia B mice receiving AAV1 hFIX muscle directed gene transfer. Despite limited *in vivo* persistence of the adoptively transferred cells, a sustained suppression lasting 10 weeks was observed. This was attributed to the emergence of antigen-specific suppression *via* the induction of endogenous Treg, which was facilitated by the transplanted Treg (Figure 2A). It has been shown that *ex vivo* expansion improves the suppressive properties of polyclonal Tregs, rendering them functionally superior to freshly isolated Tregs (145). Expanded Tregs highly upregulate CTLA-4 expression, which competes with the costimulatory molecule CD28 for binding to CD80/86 on APCs. Suboptimally activated APCs facilitate the induction of iTreg cells (57, 146).

Although cell therapy with expanded polyclonal Tregs has many advantages, it has been demonstrated that antigen-specific Tregs are more potent at 10- to 100-fold lower frequencies (147). One way to recover a sufficient number of antigen-specific Tregs is to expand them in the presence of alloantigens through a process of indirect allospecificity. This has been successfully used to promote transplantation tolerance, by expanding the recipient’s Treg pool toward donor antigens (148–151). In some cases, Treg expansion and therapeutic potential were improved by the addition of IL-2 and IL-12 (152). However, it is unclear whether it would be possible to isolate rare antigen-specific Tregs to supplement gene therapy, especially in the case of inherited protein deficiencies, where the antigen is not expressed and central tolerance may not be achieved.

## Therapy with Genetically Modified Cells

More recently, the applicability of gene therapy has moved beyond gene correction to a wider spectrum of diseases. Gene-modified cells, such as CAR-modified T cells for the eradication of hematologic cancers, have achieved breakthrough success in clinical trials (153–156). Glaxo Smithkline has introduced the first *ex vivo* stem cell gene therapy to treat patients with ADA-SCID, Strimvelis, which received approval from the European Medicines Agency in 2016 (157). Zalmoxis, a donor cell-derived T cell therapy used for H-SCT, is also poised for the market. These novel and successful trials are making way for other cutting-edge technology, such as the development of gene-editing techniques using CRISPR-Cas to increase the stability of CAR-T cells (NCT03166878) or for treating hematological malignancies in patients with HIV (NCT03164135).

Gene modification to increase antigen specificity has been recently applied to Tregs. The difficulty of isolating cells with a rare antigen specificity from the natural polyclonal T cell repertoire has hampered the clinical translation of targeted therapy with antigen-specific Tregs. On the other hand, treatment with polyclonally expanded Tregs requires the infusion of large numbers of clinical-grade autologous cells, with a possibility for general immunosuppression. Using clinical-grade LVs to genetically reprogram cells represents an attractive strategy to fine-tune Treg populations for a particular specificity (Figure 2B). One example is the ectopic overexpression of FoxP3 in conventional CD4^+^ T cells from healthy donors, with the aim of generating a large number of homogeneous and functional Treg cell populations. This technique has been applied successfully to conventional CD4^+^ T cells of patients with immune dysregulation, polyendocrinopathy, enteropathy, X-linked (IPEX) syndrome (dysfunction in FoxP3 gene), and in other preclinical animal models of autoimmunity (158–161). The possibility of reversion to an effector T cell phenotype is a concern, given the plastic nature of many Treg populations. However, adoptively transferred, FoxP3 overexpressing Tregs were shown to be stable in steady-state and inflammatory conditions and continued to be suppressive *in vivo* (161). The requirement for antigen specificity of FoxP3 gene-transferred cells and the dose of cells required for suppression, as well as persistence *in vivo*, are questions that still need to be addressed.

Another similar approach for engineering Treg specificity using TCR gene transfer has been shown to improve Treg potency, as observed in preclinical models for diabetes, transplantation tolerance, arthritis, and hemophilia A (61, 162–165). Engineered TCRs provide a viable alternative to redirect Treg specificity to a single antigenic epitope with a potentially high TCR affinity. However, this approach is HLA restricted and thus limits the number of patients to those with common HLA alleles.

Inspired by the clinical success of using CAR-T cells to treat certain types of cancers, a similar approach has been applied that engineers Tregs to express extracellular single-chain antigen-binding domains (scFv) fused to intracellular signaling molecules (Figure 2C). CARs can directly recognize their corresponding antigen irrespective of HLA. Further, issues such as TCR chain mispairing, which is a potential concern with TCR gene transfer, do not arise. At present, it is unclear how CAR-Tregs exert their suppressive effect and which cell populations they interact with. It has been postulated that the optimal activation of CAR-Tregs requires the presence of APCs (62). It is possible that CAR-Tregs recognize antigen that is immobilized on the surface of the APC, although molecular interactions or receptors that may be involved remain to be defined. The ability of CAR-Tregs to respond directly to soluble antigen or to recognize antigen bound to a B-cell receptor (BCR) is also still an open question (Figure 2C).

Chimeric antigen receptor regulatory T cells are being tested in preclinical models of EAE, allograft rejection, colitis, rheumatoid arthritis, and hemophilia A (62, 166–174). The first CAR-Treg trial, by the French company TxCell, for the prevention of transplant rejection is expected to commence in 2018. Such clinical trials will be able to address questions such as immunogenicity of the novel CAR molecule (175), or the possibility of cytokine release syndrome, which is a serious side effect of CAR-T cell treatments for cancer (176). Meanwhile, new CAR strategies are being developed to improve the specificity and function of CAR-modified T cells/Tregs. For example, the transient expression of a CAR construct that recognizes the FITC molecule can be used to target Treg function to transplanted organs by binding to FITC-conjugated monoclonal antibodies against donor MHC antigens (174). Alternatively, the surface expression of the antigenic domain, rather than the scFv, conjugated to primary and secondary signaling molecules, can bind the BCR of the corresponding antigen-specific B-cell, thus promoting B-cell depletion or suppression, as demonstrated in a model for autoimmunity and hemophilia A (177, 178).

## Challenges and Future Directions

Beginning with the discovery in 1990 and 1995 that adoptively transferred CD4^+^CD25^+^ Tregs can maintain tolerance in an autoimmune animal model (179), the clinical prospects of Tregs have expanded in the past decade (36). It is apparent from studies with disease models and clinical trials that Treg-suppressive mechanisms can counter immune activation caused by gene replacement therapy.

Although clinical trials using adoptively transferred Tregs to supplement gene therapy have not been attempted thus far, this review highlights several benefits for combining these two approaches. For example, existing obstacles faced in recent clinical trials such as unwanted immune responses to gene therapy and the inability to readminister vector could be mitigated by the codelivery of Treg with the vector. Adoptive immunotherapy with Tregs has shown clinical efficacy in autoimmune diseases such as T1D (which is characterized by a detrimental inflammatory response) and can tolerize against inflammatory reactions to a transplanted organ. We therefore propose that augmenting gene transfer applications, either by promoting the *in vivo* induction and expansion or Tregs or by immunomodulation with adoptively transferred Tregs can work synergistically and lead to successful gene transfer.

It is crucial, however, to emphasize the importance of good manufacturing practice-compliant cell therapy procedures, especially for the generation of polyclonal Tregs, which require dosing at larger cell numbers to reach therapeutic efficacy (180). A current challenge with using Tregs in the clinic is the need for the isolation and expansion of a pure population of functional and stable cells in sufficient numbers. FoxP3 is an intracellular marker and can be transiently expressed by activated CD4^+^ and CD8^+^ T cells. Optimization of cell sorting, such as employing double sorting of CD4^+^CD25^hi^CD127^lo^ cells can ensure increased purity of the starting population to help control for the outgrowth of effector T cells, which expand exponentially faster than Tregs in culture.

Regulatory T cell infusion may be beneficial not only in gene replacement settings to suppress capsid and transgene-specific immune responses but may also have the potential as adjunct therapy to prevent immune responses toward vector readministration. The formation of neutralizing antibodies constitutes a major obstacle to vector readministration, as they are elicited at high titers following gene transfer and can persist for years. Repeat administration of vector may be required when the gene product does not reach therapeutic levels, or when administered to pediatric patients, where an increasing organ turnover may limit the therapeutic dose over time. Successful gene therapy would expose the patient’s immune system to the newly delivered vector and/or transgene, generating B and T cell responses that would limit the ability to readminister vector. Plasmapheresis and transient immunosuppression (anti-CD40 Ab, CTLA-4 Ig, high-dose corticosteroids, rapamycin, rituximab, or combinations of these treatments) are currently being tested to allow for repeat injections (NCT02240407) (76, 113, 181). The use of adoptive Treg therapy in these scenarios has not been tested, and thus it remains a possible combinatorial therapy product for blocking potential immune responses.

## Author Contributions

MB, SK, CT, and RH made substantial contributions to the concept of this work, drafted, and made critical revisions and final approval. MB, SK, CT, and RH agreed to be accountable for all aspects of the work.

## Conflict of Interest Statement

The authors declare that the research was conducted in the absence of any commercial or financial relationships that could be construed as a potential conflict of interest.
